# P02.128. The effects of guided imagery on preoperative anxiety and pain management in patients undergoing Laparoscopic Cholecystectomy in a multi-centre RCT study

**DOI:** 10.1186/1472-6882-12-S1-P184

**Published:** 2012-06-12

**Authors:** M Jong, A Pijl, H de Gast, M Sjöling

**Affiliations:** 1 Louis Bolk Institute, Nutrition & Health, Driebergen, Netherlands; 2 Slotervaart Hospital, Amsterdam, Netherlands; 3 Rode Kruis Hospital, Beverwijk, Netherlands; 4 Mid Sweden University, Sundsvall, Sweden

## Purpose

Laparoscopic Cholecystectomy (LC) is common practice in treatment of symptomatic gall stones. LC is often associated with preoperative anxiety and stress which may negatively impact postoperative pain perception and recovery from surgery. The aim of the present study was to investigate whether a “non-pharmacological” intervention with guided imagery can reduce preoperative anxiety, postoperative pain perception and medication compared to standard care in patients undergoing LC.

## Methods

In a pragmatic multi-centre randomized controlled study 140 patients were randomized to a Guided Imagery (GI) group or control group. The GI group was provided with a CD to practice guided imagery once a day, 7 days prior to surgery. Patients in the control group received standard care instructions only. Primary outcome measurement was the use of postoperative analgesics. Secondary outcome parameters were preoperative anxiety levels using the Amsterdam Preoperative Anxiety and Information Scale (APAIS), postoperative pain perception (VAS-scale), general patient satisfaction (PSQ) and safety (adverse events) with treatment.

## Results

95 out of 140 randomized patients completed the study, 43 in the GI and 52 in the control group. The major reasons for dropping out were acute LCs or cancellation of LC. Both groups were highly comparable with respect to demographic data. The majority was female (GI: 77%, Control: 75%). Postoperative morphine use was not significantly different between the GI (15.8±18.5 mg) and control group (12.5±13.6 mg, p=0.34). No significant differences were observed in anxiety and postoperative VAS scores. Twenty-three percent of patients did exercises 1-3 times, 65% 4-7 times and 12% >7 times. Within GI group analysis showed significantly less postoperative morphine use upon better compliance to GI exercises (p=0.02).

## Conclusion

It is not as simple as replacing a pill with a CD. Guided Imagery seems to reduce postoperative pain medication once compliance to imagery exercises is achieved.

